# Heme detoxification by heme oxygenase-1 reinstates proliferative and immune balances upon genotoxic tissue injury

**DOI:** 10.1038/s41419-019-1342-6

**Published:** 2019-01-25

**Authors:** Andreas Hedblom, Seyed M. Hejazi, Giacomo Canesin, Reeham Choudhury, Khalid A. Hanafy, Eva Csizmadia, Jenny L. Persson, Barbara Wegiel

**Affiliations:** 1 Department of Surgery, Cancer Research Institute and Transplant Institute, Beth Israel Deaconess Medical Center, Harvard Medical School, Boston, MA USA; 2 Department of Translational Medicine, Lund University, Lund, Sweden; 3 Department of Molecular Biology, Umea University, Umea, Sweden; 4 Department of Neurology, Beth Israel Deaconess Medical Center, Harvard Medical School, Boston, MA USA

## Abstract

Phenotypic changes of myeloid cells are critical to the regulation of premature aging, development of cancer, and responses to infection. Heme metabolism has a fundamental role in the regulation of myeloid cell function and activity. Here, we show that deletion of heme oxygenase-1 (HO-1), an enzyme that removes heme, results in an impaired DNA damage response (DDR), reduced cell proliferation, and increased cellular senescence. We detected increased levels of p16^INK4a^, H2AXγ, and senescence-associated-β-galactosidase (SA-β-Gal) in cells and tissues isolated from HO-1-deficient mice. Importantly, deficiency of HO-1 in residential macrophages in chimeric mice results in elevated DNA damage and senescence upon radiation-induced injury. Mechanistically, we found that mammalian target of rapamycin (mTOR)/S6 protein signaling is critical for heme and HO-1-regulated phenotype of macrophages. Collectively, our data indicate that HO-1, by detoxifying heme, blocks p16^INK4a^ expression in macrophages, preventing DNA damage and cellular senescence.

## Introduction

Macrophages (Mφ) play a fundamental role in eliminating invading pathogens, transformed cells via phagocytosis, coordinating immune responses through cytokine expression, and producing ROS^1^. Moreover, residential Mφ maintain homeostasis by scavenging debris of apoptotic and necrotic cells^2^. However, when residential Mφ are exposed to apoptotic cells repeatedly, as they are in cancer patients undergoing chemo- or radiation therapy, their clearance of these senescent cells becomes impaired^3^. Further, residential Mφ function in immunosurveillance against senescent cells within the tissues under pathological conditions such as during organ damage^4^.

Alterations in cytokine levels in the tissue microenvironment driven by injury or bacterial infection can lead to a senile (“senescent”) phenotype of Mφ. Senile Mφ are predominantly in an active, pro-secretory state, partially due to elevated NFκB signaling. Senescence-associated secretory phenotype (SASP) is defined by production of CCL2/MCP1, TNFα, IFNγ, IL-6, growth and differentiation factors (TGFβ and HGF), and matrix remodeling enzymes (metalloproteinases; MMP1/3/10/13) and is implicated in cancer growth and organ regeneration^5,6^. Induction of a senile phenotype in Mφ as in other cells occurs in part due to the accumulation of DNA breaks following multiple rounds of ROS bursts. DNA damage and cell cycle inhibition through high expression of p16^INK4a^, p21, and p53 are the key contributors to the onset of senescence^7^.

p16^INK4a^ is involved in replicative senescence, but also controls inflammatory responses. It has been demonstrated that deficiency in p16^INK4a^ promotes the M1 pro-inflammatory phenotype of Mφ^8^. p16^INK4a^ suppressed LPS-driven inflammatory cytokine (IL-6) production in Mφ independently of cyclin-dependent kinases 4/6 (CDK4/6)^9^. mTOR, a direct target of PI3K-Akt signaling induces expression of p16^INK4a^, p21^CIP1^, and p15^INK4b^ to support cell senescent phenotype^10^. mTOR blockade is associated with suppressed senescence and SASP^11^. Indeed, rapamycin, an mTOR inhibitor, inhibits senescence-associated phenotype of cells in part via activation of Nrf2 signaling^12^, which is a direct regulator of HO-1 expression^13^. We have previously reported that mTOR signaling is induced by biliverdin (BV)^14^. Interestingly, rapamycin was shown to activate HO-1 in smooth muscle cells and suppress their growth^15^. HO-1 catalyzes the first step of heme degradation to BV, iron, and carbon monoxide (CO)^16^. Inhibition of HO-1 in endothelial cells triggered senescence^17^ and HO-1 was suggested to act as an anti-aging molecule^18^. Recent studies by Even B et al. suggest that induction of HO-1 in lung fibroblasts blocks senescence phenotype by improving mitochondria function and diminishing ROS levels^19^. Similarly, the second enzyme of the heme degradation pathway, biliverdin reductase A (BVR-A), which reduces BV to bilirubin has been shown to protect against senescence^20^.

The physiological role of the heme degradation pathway is required for proper function of immune cells and those exposed to oxidative stress^21,22^. Lack of HO-1 results in accumulation of toxic heme and initiates reactive oxygen species (ROS)-driven responses leading to cellular dysfunction. Analyses of HO-1 null mice (*Hmox1*^−/−^) and the reported phenotype of an HO-1 deficient patient support the paradigm that HO-1 is critical in cellular defense from oxidative stress, inflammation, and maintenance of cellular homeostasis^23,24^. *Hmox1*^−/−^ mice exhibit low birth/death in utero ratio and markers of high oxidative stress with impaired responses to inflammatory stimuli. Further, these mice show high tissue injury, anemia, DNA damage and a chronic inflammation similar to the human HO-1-deficient phenotype^23,24^. Significantly, bone marrow transplant (Tx) from the wild type donor mice that restored HO-1-expression in hematopoietic lineages reversed the *Hmox1*-deficient phenotype^25^. This includes severe anemia and intravascular hemolysis with damage to endothelia and kidneys^25^. Recent data suggest the role of recipient HO-1 in macrophages in the model of liver transplant^26^. Application of exogenous CO improved the outcome of septic shock and rescued mice with myeloid-specific deletion of HO-1 from bacterial sepsis^27,28^. Along with CO, the bile pigments generated by HO-1 activity possess strong anti-oxidant properties as bilirubin (BR) scavenges ROS^29^, while CO induces protective preconditioning by mild and transient induction of ROS^30^. Exogenous applications of CO or BR or biliverdin (BV), via different mechanisms, re-impose homeostasis in inflammation models;^31^ however, a detailed mechanism by which heme degradation enzymes and metabolites act in regulating Mφ functions is largely unknown.

In this study, we describe a novel role for HO-1 in modulating immune function as well as cellular senescence in response to DNA damage stimuli. Further, we demonstrate that heme induces p16^INK4a^, senescence, and DNA damage in Mφ, fibroblasts and epithelial cells. We conclude that HO-1, by removing heme, restores tissue equilibrium by preventing senescence, decreasing DNA damage, and improving immune responses.

## Results

### Lack of HO-1 induces p16^INK4a^ expression in multiple cell types and is critical for cell cycle and macrophage function

**Fig. 1:**
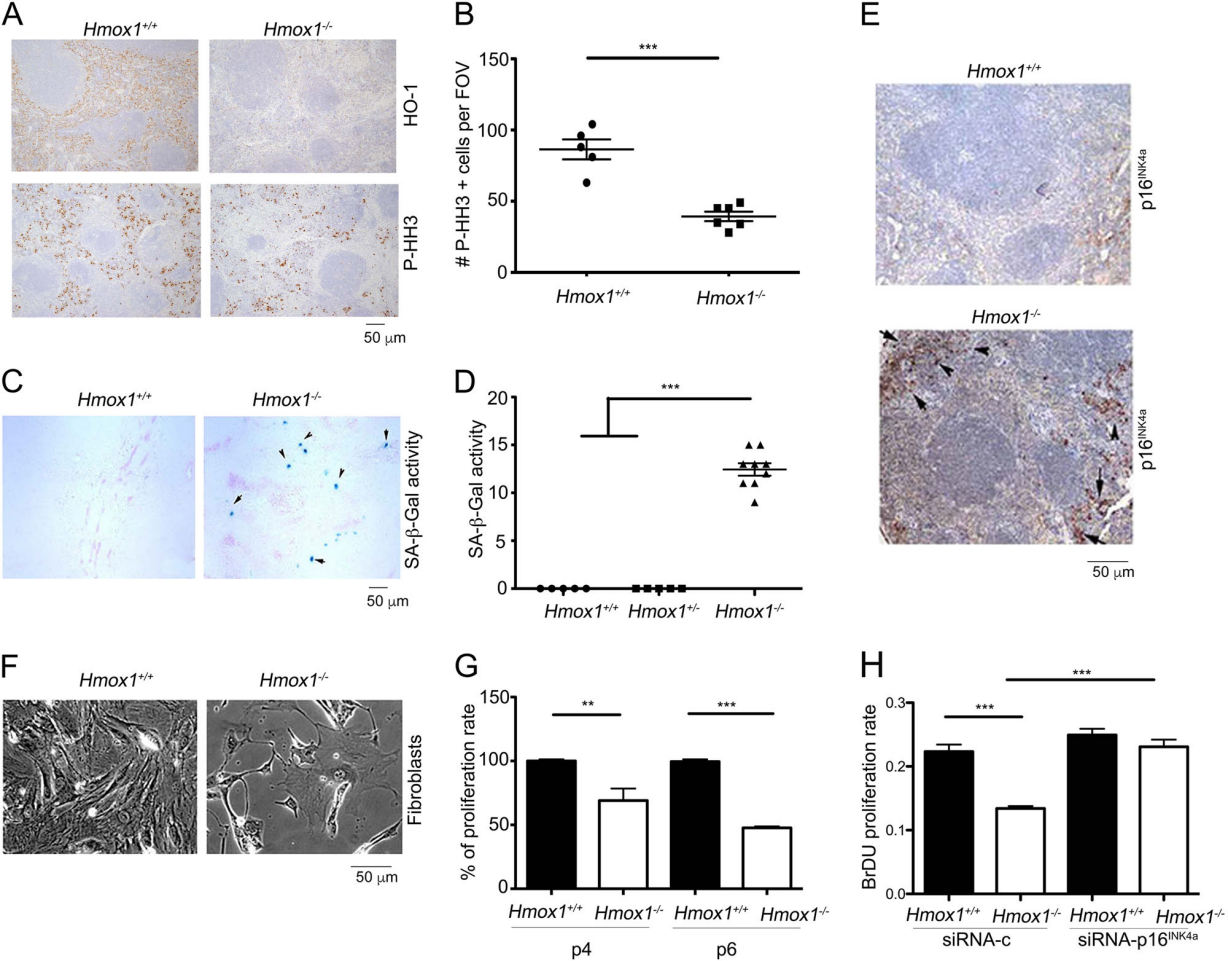
Accelerated senescence in tissues and fibroblasts isolated from *Hmox1*^−/−^ mice. **a**, **b** Spleens from *Hmox1*^+/+^ and *Hmox1*^−/−^ mice were stained with HO-1 and P-HH3 antibodies. Quantification is shown in **b**. ***p < 0.001. *n* = 5–6 fields of view (FOV) from *n* = 3 mice per group. **c**, **d** Spleens from *Hmox1*^+/+^*, Hmox1*^+/−^, and *Hmox1*^−/−^ mice were stained with X-Gal at pH 6.0 to evaluate senescence-associated β-galactosidase (SA-β-Gal) activity. Representative pictures of spleens from *Hmox1*^+/+^, *Hmox1*^+/−^, and *Hmox1*^−/−^ mice are shown in **c** and quantitation of SA-β-Gal-stained sections is presented in **d** as number of positive cells per FOV. Ten FOV were selected at random from *n* = 3 mice/group. ***p < 0.001. **e** Immunohistochemical staining with antibodies against p16^INK4a^ in the spleens from *Hmox1*^+/+^ and *Hmox1*^−/−^ mice. **f**, **g** Inhibition of proliferation of fibroblasts isolated from *Hmox1*^−/−^ mice. Representative pictures of fibroblast morphology at p6 are shown in **f**. BrdU incorporation was used to assess the proliferation rate of fibroblasts isolated from *Hmox1*^+/+^, and *Hmox1*^−/−^ animals. Results represent mean ± SD. ***p < *0.001*. **p < 0.01. **h** Proliferation of mouse fibroblasts transfected with scramble siRNA or siRNA against p16^INK4a^. Cells were transfected with siRNA for 48 h and then seeded for BrdU proliferation assay. **p < 0.01 *Hmox1*^−/−^ versus *Hmox1*^+/+^ mice

Since HO-1 deficient (*Hmox1*^−/−^*)* mice are characterized by increased DNA damage and inflammation^23^, we reasoned that these mice could exhibit abnormal changes in their tissues due to cellular senescence. Lack of HO-1 in splenocytes resulted in significantly lower phosphorylation of histone H3, a marker of cellular proliferation (Fig. 1a, b). In the same tissues, *Hmox1*^−/−^ mice showed increased activity of senescence-associated-β-galactosidase (SA-β-Gal), a marker associated with senescence (Fig. 1c, d) and p16^INK4a^ expression (Fig. 1e) compared to wild type mice. Since senescence limits the number of proliferative cycles and is associated with decreased cell functions, we assessed the proliferative capacity of primary cells in the absence of HO-1 by isolating mouse fibroblasts from *Hmox1*^−/−^ and *Hmox1*^+/+^ mice and following their replication at passage 4 and 6. Fibroblasts from *Hmox1*^−/−^ mice showed limited proliferation at passage 4 and 6, suggestive of replicative senescence (Fig. 1f, g). We also found that fibroblasts isolated form *Hmox1*^−/−^ mice show significantly upregulated p16^INK4a^ levels (Supplementary Fig. 1A) without significant changes in other cell cycle inhibitors, such as p21 (Supplementary Fig. 1B). Further, p27 was slightly lower in *Hmox1*^−/−^ mice (Supplementary Fig. 1C). To assess if p16^INK4a^ is important for replicative senescence in the absence of HO-1, we transfected fibroblasts isolated form *Hmox1*^−/−^ mice with siRNA against p16^INK4a^ or scramble siRNA (Supplementary Fig. 1D, Fig. 1h). We showed that inhibition of p16^INK4a^ by siRNA reversed the senescence-associated replicative blockade in cells lacking HO-1 (Fig. 1h).

These data indicate that HO-1 is required for modulation of p16^INK4a^ in replicative senescence of fibroblasts.

### Lack of HO-1 in macrophages or excess heme leads to alterations in DDR and mTOR-p16^INK4a^ signaling

**Fig. 2:**
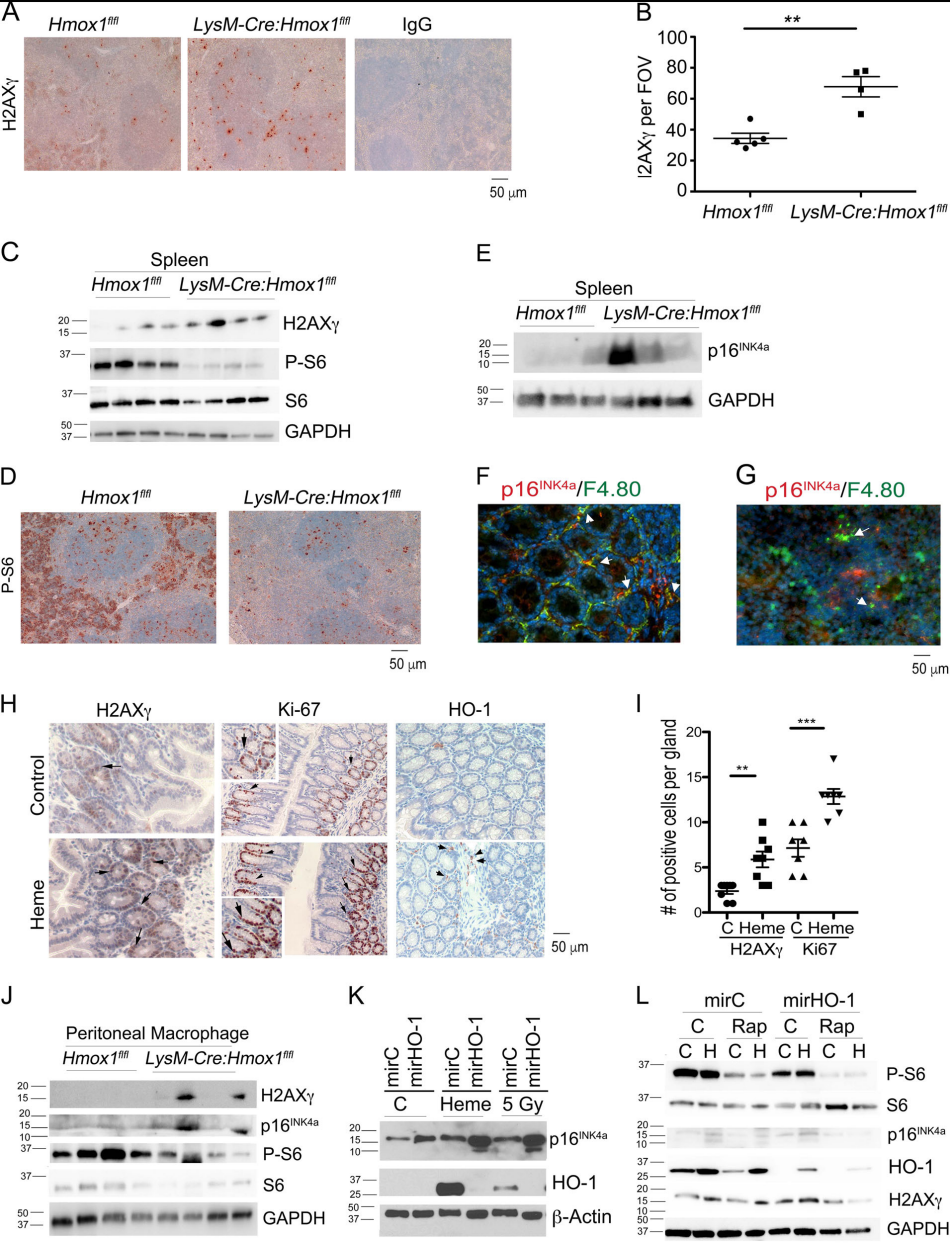
Lack of HO-1 in myeloid cells leads to low phosphorylation of S6 and high expression of p16INK4a. **a**, **b** Immunohistochemical staining with antibody against H2AXγ in the spleens from *LysM-Cre:Hmox1*^*fl/fl*^ or *Hmox1*^*flfl*^ mice. Quantification of the staining from *n* = 4–5 mice is shown in **b**. **c** Immunoblotting of the lysates of the spleens from *LysM-Cre:Hmox1*^*fl/fl*^ or *Hmox1*^*flfl*^ mice. *n* = 4 per group. **d** Immunohistochemical analysis of the phosphprylated S6 (P-S6) in the spleens from *LysM-Cre:Hmox1*^*fl/fl*^ or *Hmox1*^*flfl*^ mice. *n* = 4–5 per group. **e** Immunoblotting of the lysates of the spleens from *LysM-Cre:Hmox1*^*fl/fl*^ or *Hmox1*^*flfl*^ mice. *n* = 3 per group. **f**, **g** Immunostaining with antibodies against p16^INK4a^ and F4.80, a marker of macrophages in the colonic (**f**) or spleen (**g**) tissues of C57/Bl6 mice. **h**, **i** Immunohistochemistry with antibodies against H2AXγ, Ki67 and HO-1 in the colon of mice treated with vehicle (control) or heme (35 mg/kg, i.p.) daily for 2 weeks. *n* = 4 mice per group. Representative sections are shown in **h** and quantification in **i** *p < 0.05, **p < 0.01, ***p < 0.001. **j** Peritoneal macrophages were isolated from *LysM-Cre:Hmox1*^*fl/fl*^ or *Hmox1*^*flfl*^ mice and lysated. Western blot was performed using *n* = 4 per group. **k** RAW 264.7 macrophages with stable knockdown of HO-1 were treated with heme (50 μM) or irradiation (5 Gy) for 6 h. Immunoblotting was performed using anti-HO-1 and anti-p16^INK4a^ antibodies. Western blot is representative for *n* = 3 independent experiments. **l** RAW 264.7 macrophages with stable knockdown of HO-1 were treated with rapamycin (20 nM) for 15 min prior addition of heme (50 μM) for 6 h. Western blot is representative for *n* = 3 experiments

Replicative senescence may be initiated by irreversible DNA damage^32^. Our previous data indicated that the complete deletion of HO-1 resulted in chronic DNA damage responses (DDR) and presence of unrepaired DNA foci^33^. Since Mφ are the primary cells expressing HO-1 in the spleen, where the major DNA damage and senescence were observed in *Hmox1*^−/−^ mice, we asked whether deletion of HO-1 in macrophage (*LysM-Cre:Hmox1*^*flfl*^) may lead to a similar phenotype. Remarkably, we found that mice deficient in HO-1, specifically in myeloid cells, showed signs of DDR with elevated numbers of H2AXγ positive cells (Fig. 2a, b). We further confirmed the IHC staining with immunoblot, detecting a similar increase in H2AXγ in the spleens isolated from *LysM-Cre:Hmox1*^*flfl*^ mice (Fig. 2c). Interestingly, in these mice, we found limited activation of mTOR-S6 pathway, an upstream regulator of senescence and cell growth (Fig. 2c). We observed diminished phosphorylation of S6 in the spleens isolated from *LysM-Cre:Hmox1*^*flfl*^ mice, which correlated with increased DDR (Fig. 2c, d). We detected elevated levels of p16^INK4a^ in the same tissues indicating that Mφ-derived HO-1 may be critical for maintenance of growth and homeostasis in the spleen (Fig. 2e). p16^INK4a^ expression was detected in the F4.80^+^ macrophage population in both colon and spleen (Fig. 2f, g).

Since HO-1 is responsible for removal of heme, we assessed whether an excess of heme may influence DDR and cellular proliferation. We injected wild type mice with heme i.p. for 2 weeks daily and assessed DNA damage and proliferation by H2AXγ and Ki67 staining, respectively. We found high levels of H2AXγ and increased Ki67 expression in the colon of heme treated mice compared to control animals (Fig. 2h-i). We focused on the colon as there is a high baseline proliferative demand in this tissue. This suggests that heme influences proliferation and DNA damages in colon in mice.

Since Akt-mTOR-S6 pathway has been previously linked to DDR, senescence and abnormal cellular phenotype^34^, we isolated peritoneal macrophages (PM) from *Hmox1*^*flfl*^ and *LysM-Cre:Hmox1*^*flfl*^ mice to assess their baseline levels of H2AXγ and mTOR pathway (Fig. 2j). We found that PM isolated from three out of four *LysM-Cre:Hmox1*^*flfl*^ mice showed higher levels of H2AXγ and slightly lower phosphorylation of S6 (Fig. 2j), indicating that HO-1 is important regulator of Mφ function.

Since macrophage-expressed HO-1 detoxifies the cellular environment from free heme, we next assessed the role of HO-1 in controlling macrophage phenotype in the presence of heme (Fig. 2k). We found that RAW264.7 macrophages lacking HO-1 show high levels of p16^INK4a^ (Fig. 2k). The levels of p16^INK4a^ were elevated by heme treatment and p16^INK4a^ expression was further amplified in cells lacking HO-1 (Fig. 2k). Importantly, heme-induced p16^INK4a^ expression was controlled in part by mTOR signaling, as rapamycin inhibited phosphorylation of S6 and decreased heme-induced p16^INK4a^ expression (Fig. 2l). Interestingly, rapamycin also inhibited HO-1 expression, suggesting a negative feedback loop on the regulation of heme-induced p16^INK4a^ levels (Fig. 2l). There was no effect of rapamycin on heme-induced H2AXγ (Fig. 2l). Similarly, in the primary bone-marrow derived macrophages (BMDM) isolated from *LysM-Cre:Hmox1*^*flfl*^ mice and treated with heme or hydrogen peroxide for 8 h, there was increased phosphorylation of S6 protein (Supplementary Fig. 2A). These effects were blocked by rapamycin. BMDM from *LysM-Cre:Hmox1*^*flfl*^ mice had lower P-S6 levels in response to heme or H_2_O_2_ (Supplementary Fig. 2A). We showed increased levels of p16^INK4a^ and HO-1 in response to heme or hydrogen peroxide treatment. These effects were dependent on mTOR signaling. Interestingly, heme or hydrogen peroxide-induced DNA damage as measured by H2AXγ was not altered by rapamycin (Supplementary Fig. 2A). Furthermore, higher p16^INK4a^ and H2AXγ in response to heme treatment in BMDM from *LysM-Cre:Hmox1*^*flfl*^ mice corresponded to lower number of cells as measured after 24 h (Supplementary Fig. 2B), suggesting poor proliferation and/or survival of BMDM isolated from *LysM-Cre:Hmox1*^*flfl*^ mice compared to control mice.

These data suggest that inefficient detoxification of heme may lead to the abnormal expression of p16^INK4a^ and H2AXγ, which may contribute to the fragility of BMDM isolated from *LysM-Cre:Hmox1*^*flfl*^ mice.

### Deletion of HO-1 in residential macrophages in the colonic tissues results in chronic DDR and senescence

**Fig. 3:**
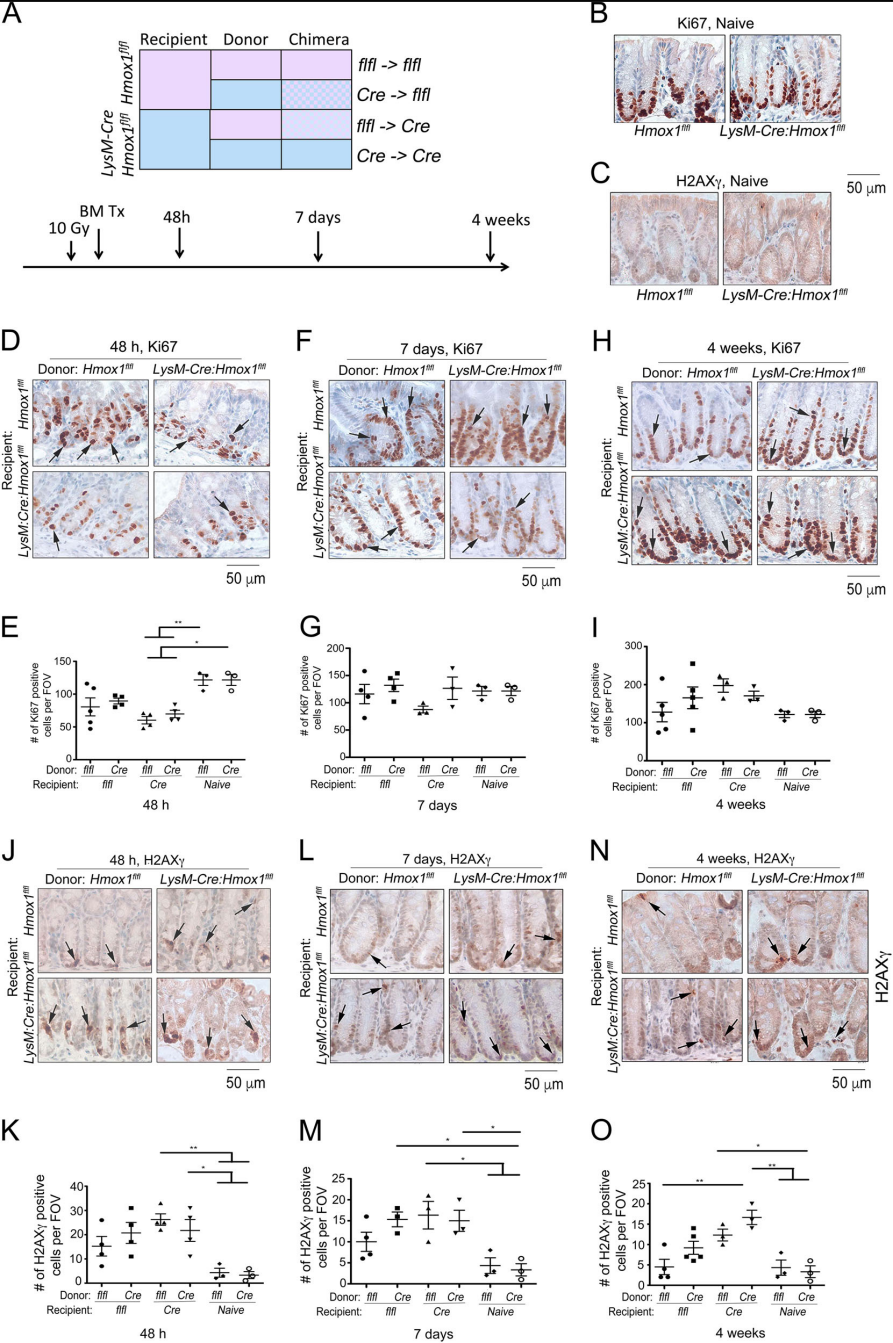
Lack of HO-1 in residential macrophages in colons results in poorer recovery after genotoxic stress. **a** A scheme illustrating generation of chimeric mice and harvest time points. **b** Immunohistochemical staining of Ki67 and H2AXγ in naïve *Hmox1*^*flfl*^ and *LysM-Cre:Hmox1*^*fl/fl*^ mice. **c**–**o** Representative pictures of staining and quantifications of number of cells positive for Ki67 (**c**–**i**) or H2AXγ (**c**, **j**–**o**) in the colon after 10 Gy irradiation followed by BM Tx from *LysM-Cre:Hmox1*^*fl/fl*^ or *Hmox1*^*flfl*^ donor to *LysM-Cre:Hmox1*^*fl/fl*^ or *Hmox1*^*flfl*^ recipient (R) mice. 48 h, 7 days or 4 weeks after BM Tx colon tissues were harvested and stained. *p < 0.05; **p < 0.01

In an effort to define the role of myeloid-derived HO-1 in the regulation of replicative senescence and Mφ dysfunction due to immunosenescence, we used chimeric mice after transplanting bone marrow from *Hmox1*^*flfl*^ or *LysM-Cre:Hmox1*^*flfl*^ mice into *Hmox1*^*flfl*^ or *LysM-Cre:Hmox1*^*flfl*^ recipient mice (Fig. 3a). In this model, lethal doses of radiation induce DDR in tissues with high replicative potential, such as colon. Although, these mice are rescued from death by bone marrow transplant, we reasoned that the lack of HO-1 in a donor or in the recipient tissue Mφ may influence the recovery processes. All mice survived the BM transplant, however *LysM-Cre:Hmox1*^*flfl*^ recipient mice transplanted with bone marrow from either *LysM-Cre:Hmox1*^*flfl*^ or *Hmox1*^*flfl*^ mice showed lower epithelial proliferation in the colon after 48 h (Fig. 3d, e) compared to naïve mice (Fig. 3c–e). *Hmox1*^*flfl*^ recipient mice transplanted with either BM recovered quickly, as Ki67 levels were not different from naïve mice (Fig. 3d, e). We further evaluated Ki67 staining at 7 days and 4 weeks after BM transplant and found no major statistical difference in proliferation at this time points (Fig. 3f–i). However, *LysM-Cre:Hmox1*^*flfl*^ recipient mice transplanted with bone marrow from either *LysM-Cre:Hmox1*^*flfl*^ or *Hmox1*^*flf*^ mice presented with slightly elevated Ki67 levels in the colon compared to naïve mice, which might indicate compensatory proliferation or abnormality associated with poor repair (Fig. 3h, i). Interestingly, at each time points tested *LysM-Cre:Hmox1*^*flfl*^ recipient mice transplanted with bone marrow from either *LysM-Cre:Hmox1*^*flfl*^ or *Hmox1*^*flf*^ mice had higher levels of H2AXγ, indicative of chronic, unrepaired DNA damage foci (Fig. 3j–o). Importantly, H2AXγ was statistically higher in *LysM-Cre:Hmox1*^*flfl*^ recipient mice transplanted with bone marrow from *LysM-Cre:Hmox1*^*flfl*^ mice at 4 weeks compared to *Hmox1*^*flfl*^ or naïve mice (Fig. 3o).

**Fig. 4:**
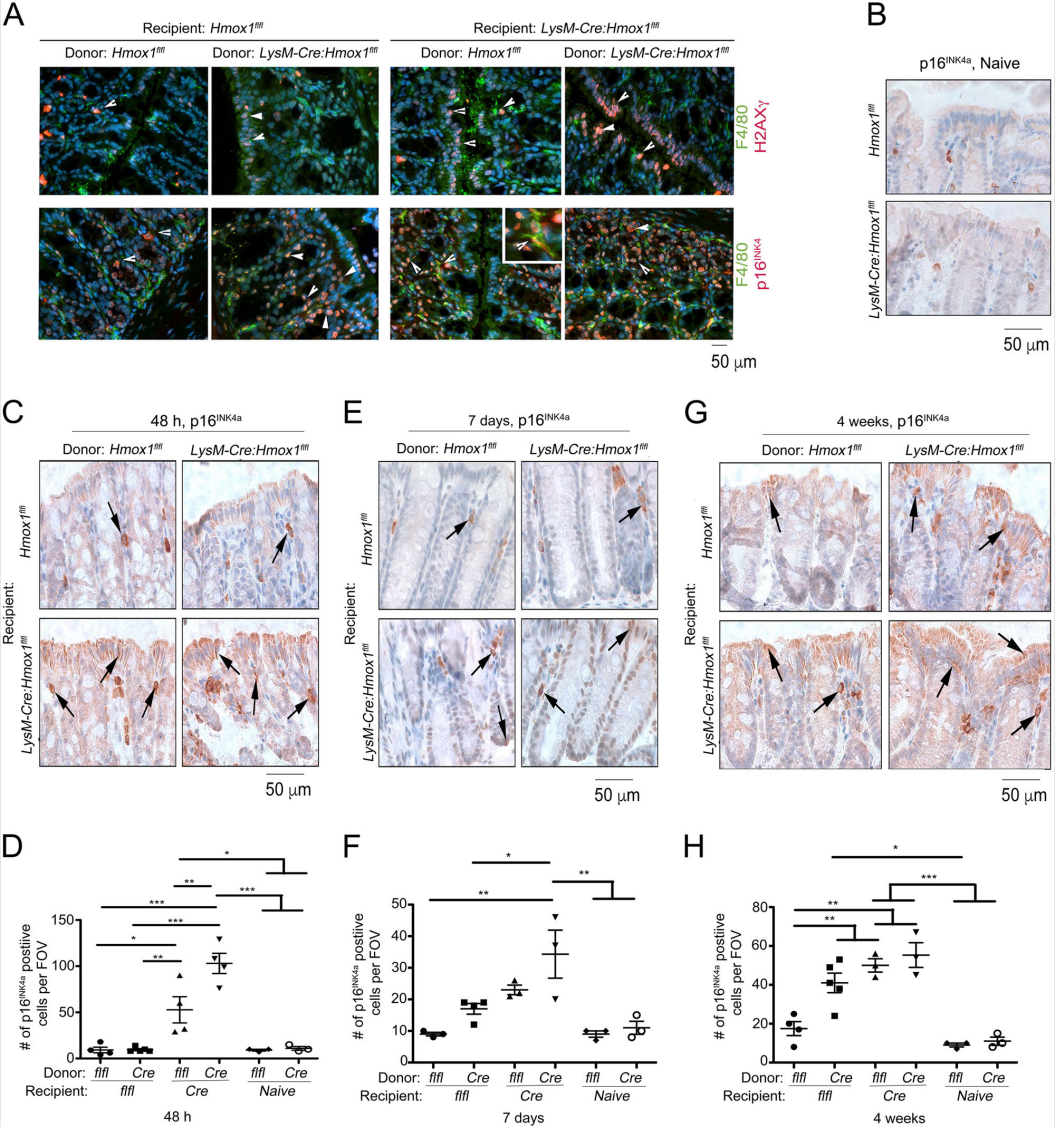
Expression of p16INK4a in the colonic epithelium in response to genotoxic stress in HO-1 chimeric mice. **a** Expression of p16^INK4a^ (red) or H2AXγ (red) in Mφ (F4.80-green) in the colon after 10 Gy irradiation followed by BM Tx from *LysM-Cre:Hmox1*^*fl/fl*^ or *Hmox1*^*flfl*^ donor (D) to *LysM-Cre:Hmox1*^*fl/fl*^ (*LysM-Cre*) or *Hmox1*^*flfl*^ (*flfl*) recipient (R) mice at 48 h after BM-Tx as in Fig. 4. Naive- non-transplanted mice. Co-localization of p16^INK4a^ and F4.80 is highlighted in inset (400x). **b** Immunohistochemical staining of p16^INK4a^ in naive colons isolated from *Hmox1*^*flfl*^ and *LysM-Cre:Hmox1*^*fl/fl*^ mice. **c**–**h** Representative pictures of staining and quantifications of number of cells positive for p16^INK4a^ in the colon after 10 Gy irradiation followed by BM Tx from *LysM-Cre:Hmox1*^*fl/fl*^ (*Cre*) or *Hmox1*^*flfl*^ (*flfl*) donor to *LysM-Cre:Hmox1*^*fl/fl*^ (*Cre*) or *Hmox1*^*flfl*^ (*flfl*) recipient (R) mice. 48 h (**c**, **d**), 7 days (**e**, **f**) or 4 weeks (**g**–**m**) after BM Tx colon tissues were harvested and stained. *p < 0.05; **p < 0.01, ***p < 0.001

Assessing the expression of p16^INK4a^ and H2AXγ in the colonic tissues, we found that colonic epithelium and F4/80-expressing macrophages were positive for p16^INK4a^. H2AXγ was primarily detected in the epithelial layer and was only found in a small amount of F4/80+ cells (Fig. 4a). To assess the role of p16^INK4a^ in chimeric mice, we stained tissues with antibody against p16^INK4a^. Naive mice showed little sign of p16^INK4a^ staining in the colonic epithelium (Fig. 4b). The major accumulation of p16^INK4a^ was observed in mice with deletion of HO-1 in residential cells transplanted with BM from *LysM-Cre:Hmox1*^*flfl*^ or *Hmox1*^*flfl*^ mice at 48 h (Fig. 4c, d) and 72 h (Fig. 4e, f) and remained positive at 4 weeks (Fig. 4g, h). Most importantly, at 4 weeks *Hmox1*^*flfl*^ chimeras transplanted with BM from *LysM-Cre:Hmox1*^*flfl*^ mice as well as recipient *LysM-Cre:Hmox1*^*flfl*^ mice transplanted with BM from *Hmox1*^*flfl*^ or *LysM-Cre:Hmox1*^*flfl*^ mice were positive for p16^INK4a^ (Fig. 4g, h) suggesting the importance of HO-1 in macrophages in long-term protection of colonic epithelium and residential Mφ after DDR event.

### The role of heme degradation products in mediating the effects of HO-1 on cellular senescence

**Fig. 5:**
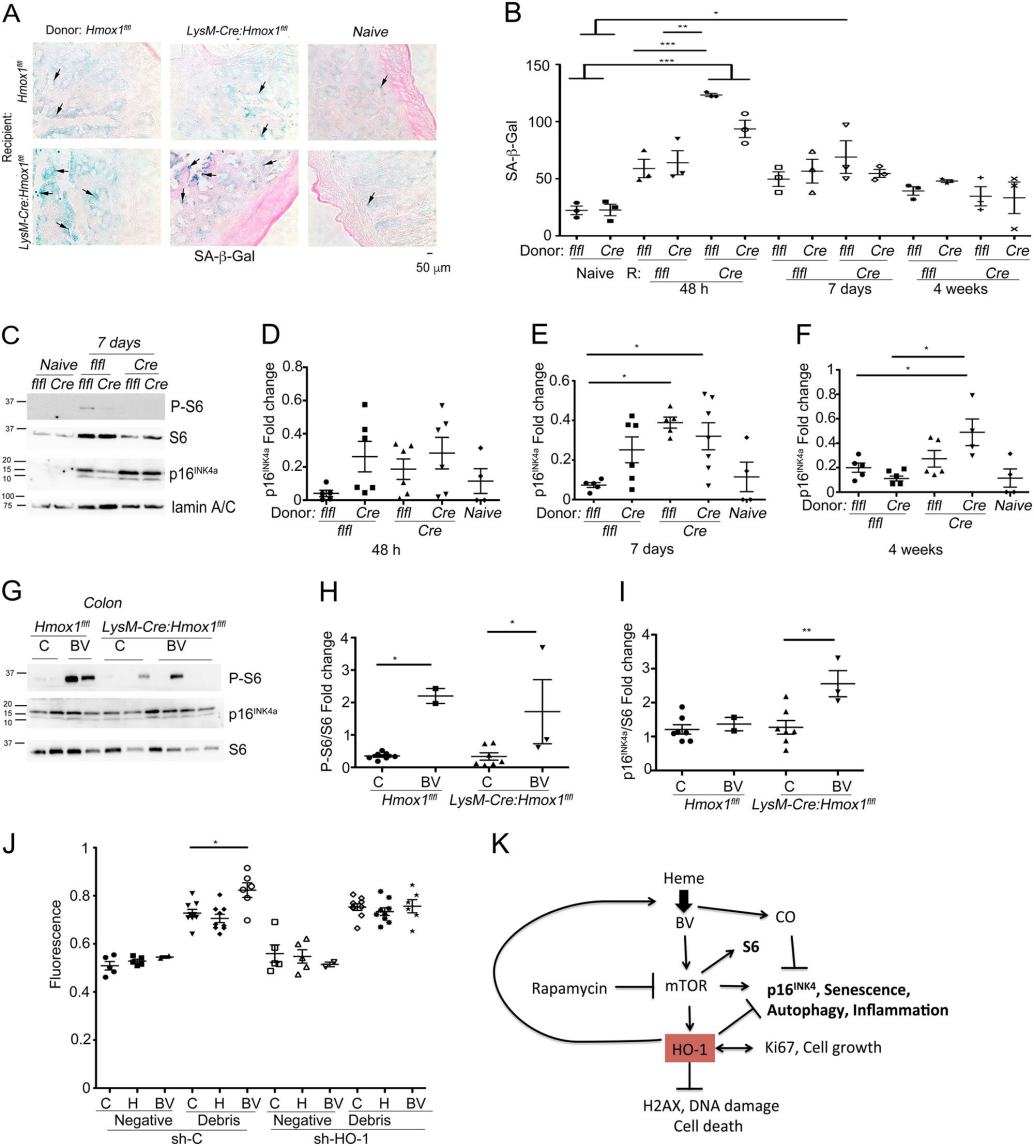
Lack of HO-1 in macrophages in the colon leads to increased senescence: role of HO-1 in BV-driven phosphorylation of S6 and uptake of necrotic cells. **a**, **b** Representative pictures of staining (48 h) and quantifications of number of cells positive for SA-β-Gal staining in the colon after 10 Gy irradiation followed by BM Tx from *LysM-Cre:Hmox1*^*fl/fl*^ (*Cre*) or *Hmox1*^*flfl*^ (*flfl*) donor to *LysM-Cre:Hmox1*^*fl/fl*^ (*Cre*) or *Hmox1*^*flfl*^ (*flfl*) recipient (R) mice. *p < 0.05; **p < 0.01, ***p < 0.001. **c**–**f** Immunoblotting of the lysates from colon tissues were harvested from chimeric mice as in **a**. **d**–**f** Shows quantification of the blots from *n* = 3–4 mice per group. *p < 0.05. **g**–**i** *Hmox1*^*flfl*^ and *LysM-Cre:Hmox1*^*fl/fl*^ mice were treated with BV (35 mg/kg, i.p.) and colon tissue were harvested at 4 h. Western blotting with antibodies against P-S6, S6, and p16^INK4a^ was performed. Quantification of the blots is shown in **h**, **i**. *n* = 3–6 mice per group. **j** Fluorescence of propidium iodide-labeled necrotic cells uptaken by RAW macrophages (mirHO-1 and control) treated with BV or heme (50 μM) for 24 h. **k** The scheme of proposed mechanism of HO-1/heme interactions with mTOR→p16^INK4a^ signaling

Lack of HO-1 in residential macrophages corresponded to the higher level of SA-β-Gal staining in the colon at 48 h, which was still slightly elevated at 7 days and subsided at 4 weeks (Fig. 5a, b). Interestingly, the total levels of p16^INK4a^ in the colon tissues were highly elevated at 7 days and 4 weeks in chimeric mice lacking HO-1 in residential macrophages as assessed by WB (Fig. 5c–f).

To dissect the mechanism behind heme/HO-1–modulated phenotype of macrophages, we treated mice with BV, a metabolite of HO-1 and regulator of Akt-mTOR signaling. Since BV was shown to regulate mTOR pathway, we asked whether BV would regulate mTOR/p16^INK4a^ signaling in the colon similarly to that observed in response to HO-1/heme modulation. We found that BV increased phosphorylation of S6 in the colons isolated from *LysM-Cre:Hmox1*^*flfl*^ or *Hmox1*^*flfl*^ mice (Fig. 5g, h). Interestingly, the effect of BV on P-S6 was sustained but lower in *LysM-Cre:Hmox1*^*flfl*^ mice. However, BV induced p16^INK4a^ only in colons isolated from mice deficient in HO-1 in Mφ (Fig. 5h, i), suggesting that the effects of BV are in part mediated through HO-1 signaling.

Since lack of proper removal of debris in the tissues is partly due to abnormal “senile” function of Mφ, we assessed whether HO-1 or BV impacts the removal of necrotic cellular debris. We did not find any significant effect on necrotic debris removal in the absence of HO-1 in RAW264.7 (Fig. 5j). However, BV-induced clearance of necrotic cells was suppressed in Mφ lacking HO-1 (Fig. 5j). This data suggest that BV effects might be in part dependent on HO-1 and that the presence of BV is important for proper scavenging of cellular debris.

These data indicate that products of HO-1 enzymatic activity are critical in regulating the expression of mTOR and p16^INK4a^. Thus, balance between the levels of heme, BV and HO-1 may protect against senescent phenotype of Mφ and possibly other cells.

## Discussion

Immunosuppression and/or aberrant immune responses constitute the most common and pernicious side effects of chemotherapy and radiation. This leads to an increased susceptibility to infection, chronic inflammation and tissue damage and may facilitate cancer recurrence. Further, immunotherapy, which is increasingly used in combination with traditional genotoxic therapies, depends on the maintenance of immune integrity. Here we describe a novel role of HO-1 in controlling senescence phenotype in macrophages and other cell types. We found an importance of residential macrophages-derived HO-1 to be required for homeostatic responses to genotoxic stress in the colon. Interestingly, HO-1 cross-talks with mTOR signaling to block p16^INK4a^ expression in response to heme (Fig. 5k). We provide a partial explanation for how the regulation of the heme pathway is involved in maintaining tissues balance.

DNA damage in the intestinal epithelial cells upon chemotherapy or radiation may be irreversible and long-lasting even after the end of the therapy. Cellular senescence occurs when the damage is irreversible and cell cycle progression is thus blocked. HO-1 has been shown to regulate cellular proliferation and apoptosis in various cell types^16,35^. We found that myeloid-derived HO-1 is critical for restoration of epithelial homeostasis in response to radio- or chemo- toxicities. HO-1 knockout mice have high levels of chronic inflammation in their organs and show signs of unrepaired DNA damage^33^. Accumulation of heme in HO-1 knockouts may be a driver of cellular dysfunction via elevated DNA damage and senescence. This includes macrophage inability to clear the bacteria^27^ or respond to sterile or pathogen-associated inflammation. We detected higher levels of p16^INK4a^ and SA-β-gal in tissues and cells isolated form HO-1 knockouts, indicating the senescent-like phenotype of these cells. In our study, knockdown of p16^INK4a^ in HO-1 fibroblasts restored their proliferative capacity, suggesting that p16^INK4a^ is a critical regulator of replicative senescence in the absence of HO-1. Replicative senescence is driven by oxidative stress. It is likely that the lack of HO-1 and thus higher heme levels leading to low duplication of fibroblasts is due to increased oxidative stress^36^. Indeed, heme-induced cell death occurs in macrophages lacking HO-1. Oxidative stress occurs during pathological conditions such as inflammation, ischemia, hyperglycemia. ROS cause DNA damage and stall in replication, which contributes to premature aging and/or cellular transformation. Inflammaging is often associated with poor removal of senescent cells that lead to spread of this phenotype on neighboring cells through SASP^37^.

Heme oxygenase-1 (HO-1) and heme degradation products are known to be protective homeostatic molecules. We have previously reported that HO-1/CO activates DNA repair via ATM-H2AXγ signaling pathway. In this study, we performed bone marrow transplants (BM Tx) using recipient mice lethally irradiated to eliminate myeloid cell. This dose of irradiation also induced DNA damage in multiple other proliferative tissues. We have shown higher levels of H2AXγ in colon in the recipients receiving transplant from *LysM-Cre:Hmox1*^*fl/fl*^ compared to control *Hmox1*^*fl/fl*^ mice. Apart from more DNA damage, the mice receiving *LysM-Cre:Hmox1*^*fl/fl*^ bone marrow also had increased expression of p16^INK4^ and less Ki67 staining, suggesting lower proliferation of epithelium. Decreased residential Mφ function or poor survival in *LysM-Cre:Hmox1*^*fl/l*^ mice is a likely contributor of the decreased repair of epithelium. The combination of impaired DNA repair and cell cycle inhibition in the organs of mice receiving bone marrow lacking HO-1 expression specifically in myeloid cells, suggests an important role for myeloid cells-derived HO-1 in recovery of gut epithelium following genotoxic treatments.

Heme deficiency causes premature senescence of neurons^38^. Senescent cells accumulate in vivo with increased age as well as at the pathological sites^39^. There are two ways how senescent cells contribute to aging: by secreting proteases and factors which disrupt tissue function and by disrupting tissue remodeling due to senescence in stem cells and progenitors^40^. HO-1 knockout mice are characterized by senescence in highly proliferative and heme-enriched tissues such as spleen but do not develop typical phenotype of progeria. However, these mice have abnormal function of their immune system. Accumulation of senescent cells may lead to chronic inflammation.

Next to two major pathways that control replicative senescence: p53 and pRb, mTOR was shown to be implicated in control of cell senescence. The locus INK4 (p19, p16, and p21) is implicated in control of senescence. We found that heme-induced p16^INK4a^ expression is highly dependent on mTOR pathway. HO-1 is required for the effects of BV on regulation of mTOR signaling. This is a novel observation of a feedback loop, in which mTOR controls the expression of HO-1 and is required for the downstream effector molecules such as p16^INK4a^ in response to heme.

Our study suggests that HO-1 derived from myeloid cells is important for recovery of the intestinal epithelium after stress associated with genotoxic cancer treatments. Increased H2AXγ may be a result of a delayed or inhibited DNA repair. Rather than an increased effort to repair, it could be interpreted as a lowered ability to get past the repair process, and instead induce senescence in these tissues. The combination of impaired DNA repair and cell cycle inhibition in the organs of mice receiving bone marrow lacking HO-1 expression specifically in myeloid cells suggests an important role for myeloid cells-derived HO-1 in recovery of gut epithelium following genotoxic treatments. Finally, our studies emphasize the role of HO-1 in residential Mφ in response to injury in the gut.

In summary, our data suggest the important role of HO-1 in controlling senescence of immune and other cells, therefore explaining in part the relevant role of heme degradation pathway in maintaining tissue homeostasis.

## Materials and methods

### Animal models and treatments

All experimental procedures were performed in accordance with relevant guidelines and regulations. All experiments were approved by the Institutional Animal Committee IACUC at BIDMC. Animals with conditional macrophage deletion of HO-1 (*LysM-Cre:Hmox*^*fl/fl*^) and control mice (*Hmox1*^*fl/fl*^) were generated as previously described^27^. *Hmox1*^+/+^ (wt) and *Hmox1*^−/−^ were previously described^41^. Mice were injected with vehicle or heme (35 mg/kg, i.p.) daily for 2 weeks and tissues were harvested for further analysis. Mice with conditional deletion of HO-1 or control mice were treated with biliverdin (35 mg/kg, i.p.) and tissues were harvested after 4 h for further analyses.

### Bone marrow transplant (BM Tx)

Mice with conditional deletion of HO-1 (*LysM-Cre:Hmox1*^*fl/fl*^) or control mice (*Hmox1*^*fl/fl*^*)* were used as donors and recipients of BM. Bones from donor animals were crushed using mortar and pestle. BM cells were isolated by flushing and aggregates were avoided by using a strainer with a 40 μm mesh. Cells were counted and re-suspended in PBS buffer at a concentration of 5 × 10^7^ cells/ml. Cells were kept on ice until injected in recipient mice.

Prior to BM Tx, recipient mice were gamma irradiated at a dose of 10 Gy in order to eliminate their bone marrow/immune cells. BM was injected via penile vein. A total of 5 × 10^6^ cells from donors were injected into each mouse under isofluorane anesthesia. All animals survived the BM Tx and their weight was regularly measured. The mice were sacrificed at 48 h, 7 days and 4 weeks after the transplant. Organs (spleen, kidney, liver, colon, intestine, BM) were collected for further analyses as described below.

### Bone marrow-derived macrophages (BMDMs) culture

Primary BM cells were isolated, differentiated and cultured as previously described^42^. Briefly, BM cells were isolated from the mouse femurs by flushing with RPMI medium (Thermo Scientific) supplemented with Antibiotic-Antimycotic solution (Life Technologies). Isolated cells were differentiated for 5 days in M-CSF medium (RPMI containing: 20 ng/ml mouse recombinant M-CSF (ProSpec), 15% fetal calf serum (FCS; Atlanta Biologicals), Antibiotic-Antimycotic solution). Fresh M-CSF-containing medium was added to cells at day 3 of culture. Where indicated, BMDM were treated with heme (50 μM) or hydrogen peroxide for 8 h prior to analysis.

### Isolation of peritoneal macrophages

Peritoneal macrophages were isolated by flushing the mouse peritoneum with 1 mL of PBS. Harvested cells were subjected to protein extraction and western blotting analysis as described below.

### Immunohistochemistry and immunofluorescent staining

Tissue samples were formalin- or Zn- fixed followed by paraffin embedding and immunostaining of 5 μm sections as previously described^42^. H&E staining was performed as reported before^43^. Sections were stained with the following antibodies: HO-1 (Enzo Laboratories); P-(Ser10)Histone H3 (Cell Signaling); p16^INK4a^ (Santa Cruz Biotechnology), P-(Ser139)-H2AX (H2AXγ) (Cell Signaling); P-S6 (Cell Signaling), Ki67 (Dako), F4/80 (Biolegend).

For immunofluorescence staining, tissues were isolated and frozen in the freezing medium using ice-cold methyl butane. Tissues were cut in 6 µm sections using a CryoTome; sections were placed on glass slides, then stored at −80 °C or immediately used for staining. Tissue sections were then fixed with 2% PFA followed by permeabilization with 0.5% Triton X-100. Sections were then incubated for 30 min in a blocking buffer containing 7% horse serum (Vector Laboratories) in PBS. A primary antibody was then applied overnight at 4 °C. Sections were then incubated with biotin-labeled secondary antibody (1.5 μg/ml in PBS; Vector Laboratories) or fluorescently labeled secondary antibodies for 1 h at room temperature. The images were captured using a Fluorescence Microscope (Zeiss). The following antibodies were used: p16^INK4a^ (Santa Cruz Biotechnology), F4/80 (Biolegend), P-(Ser139)-H2AX (H2AXγ) (Cell Signaling). For X-gal staining, spleens from *Hmox1*^+/+^ (wt) and *Hmox1*^−/−^ were stained with X-Gal at pH 6.0 to evaluate senescence-associated β-galactosidase (SA-β-Gal)) activity. Ten FOV were selected at random from *n* = 3 mice/group.

### Cell culture

Mouse fibroblasts were obtained from adult *Hmox1*^*−/−*^ and *Hmox1*^+/+^ mice. Cells were cultured between passages 4–7 in DMEM medium supplemented with 10% FBS and antibiotics. The murine macrophage cell line RAW 264.7 was grown in DMEM supplemented with 10% FBS and antibiotics. Stable HO-1 knocked-down cells (mirHO-1) and control knocked-down cells (mirC) were previously described^27^. Stable cells were selected with 1 μg/ml puromycin for 2–3 weeks and used for further analysis. Where indicated, RAW cells were pretreated with rapamycin (20 nM) and/or subsequently with heme (50 μM) for 6 h before harvesting. For irradiation experiments, RAW cells were subjected to gamma irradiation and harvested after 6 h.

### Western blotting

Cells or tissues were lysed by a freeze-thaw cycle in ice-cold lysis buffer [1% Nonidet P-40, 50 mM Tris-HCl (pH 7.5), 150 mM NaCl, 1 mM EDTA (pH 8.0), 1 mM NaF] followed by sonication in the presence of a protease inhibitor cocktail (Roche). Samples were centrifuged for 20 min at 14,000 × *g* at 4 °C, and the supernatants were collected. Protein concentration was measured using a BCA Protein Assay Kit (Pierce). For each sample, 20–50 μg of proteins were prepared in 4X NuPAGE buffer (Invitrogen) and heated to 95 °C for 5 min prior to loading on a 4–12% SDS-PAGE gel (Invitrogen). After separation and transfer of the proteins to PVDF membranes (Amersham), the membranes were blocked with 5% nonfat dry milk and probed with appropriate primary antibodies followed by HRP-conjugated secondary antibodies (Cell Signaling) at a dilution of 1:5000. Bands were visualized using SuperSignal Chemiluminescent Substrate (Pierce) exposed on ECL Film (ISC BioExpress), or CCD camera. The following antibodies were used: p16^INK4a^, P-S6 (Cell Signaling), P-(Ser139)-H2AX (H2AXγ) (Cell Signaling), HO-1 (Enzo Laboratories), β-Actin (Sigma Aldrich), lamin A/C and GAPDH (Cell Signaling Technologies).

### BrdU proliferation assay

BrdU proliferation assay kit was purchased from Roche Applied Science (Mannheim, Germany) and used according to the manufacturer’s protocol.

### In vitro viability assay

Cell viability was measured as previously described^27,44^. Briefly, cells were stained with Crystal Violet solution (Sigma-Aldrich) for 20 min at room temperature and then extensively washed in double-distilled water. Wells were dried and 10% acetic acid was added to each well to dissolve the staining. The absorbance was measured at 560 nm using an ELISA plate reader.

### Cell debris uptake

RAW 264.7 were exposed to UV radiation for 15–20 min to induce cell death. 24 h later, propidium iodide (PI) was added to the culture to label necrotic cells. Dead cells were counted and added to the RAW 264.7 cells seeded in 6 well plate at ratio 1:2. At 6 h, RAW 264.7 cells were washed 3 times with PBS and scraped in water and PI fluorescence was measured by ELISA plate reader.

### siRNA transfection

Transient siRNA transfections were performed with the Lipofectamine 2000 reagent (Invitrogen), according to the manufacturer’s instructions. The siRNA oligonucleotides were purchased from Darmacon. After 16 h, the transfection medium was replaced with fresh cell medium supplemented with 10% FBS, and the cells were subsequently allowed to grow for 48 h prior to analysis.

### RNA isolation and real time PCR

Total RNA was isolated from cultured cells using RNeasy^®^ Plus Mini Kits (Qiagen, Valencia, CA, USA) and qPCR was performed as previously described^33^. Primers for β-actin, p16^INK4a^, p27, p21 were purchased from Life Technologies and sequences are shown below. Briefly, RNA was reverse transcribed using iScript^TM^ cDNA synthesis kit (BioRad) and qPCR was performed with Mx3000P QPCR system (Agilent Technologies, Santa Clara CA). The relative quantification of gene expression was analysed using ∆ C_T_ method, normalized to the housekeeping gene and expressed as 2^−∆∆ CT^.

p16^INK4a^: F:GGAGGAAGAAAGAGGAGG, R: ACTTCGTCCTCCAGAGTCG

p27: F: AGGAGAGCCAGGATGTCAGC, R: CAGAGTTTGCCTGAGACCCAA

p21: F: AGCCTGAAGACTGTGATGGG, R: AAAGTTCCACCGTTCTCGG

β-actin: F: CCACAGGATTCCATACCCAAGA, R: TAGACTTCGAGCAGGAGATGG

### Statistical analysis

All in vitro experiments were performed at least 3 times in triplicate. All statistical analyses were performed using Graph Pad Prism software (GraphPad Prism version 5c, La Jolla, California, USA) and statistical significance was determined using ANOVA or Student’s *t*-test.

## Supplementary information

Supplementary Figure 1

Supplementary Figure 2

Supplemental figure legends
