# Supplementary figures and images for: Heme detoxification by heme oxygenase-1 reinstates proliferative and immune balances upon genotoxic tissue injury
Source: Cell Death Dis. 2019 Jan 25;10(2):72. doi: 10.1038/s41419-019-1342-6 (PMC6347604; doi:10.1038/s41419-019-1342-6)

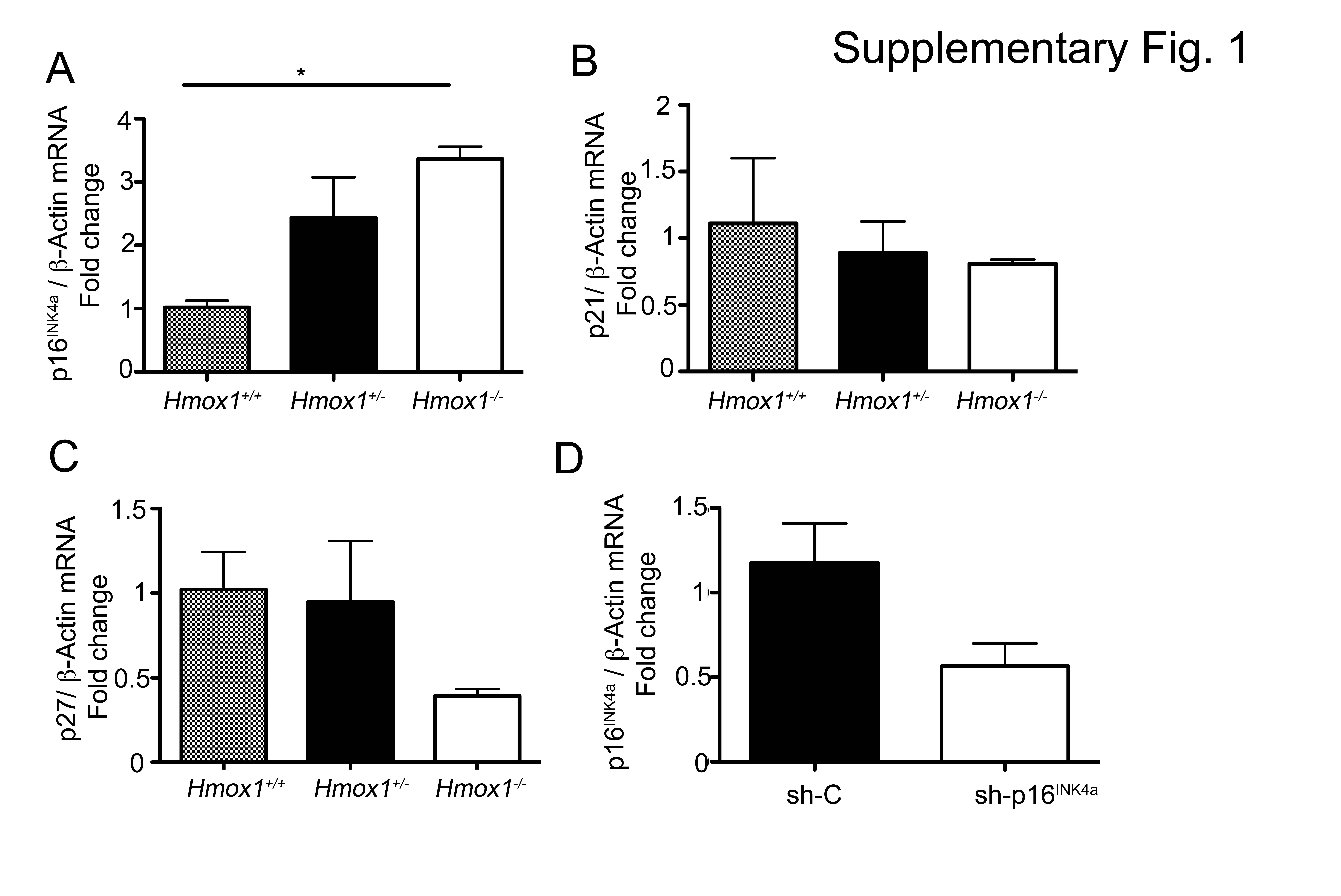

Supplement: Supplementary file 1 — Supplementary Figure 1 [file 41419_2019_1342_MOESM1_ESM.jpg]

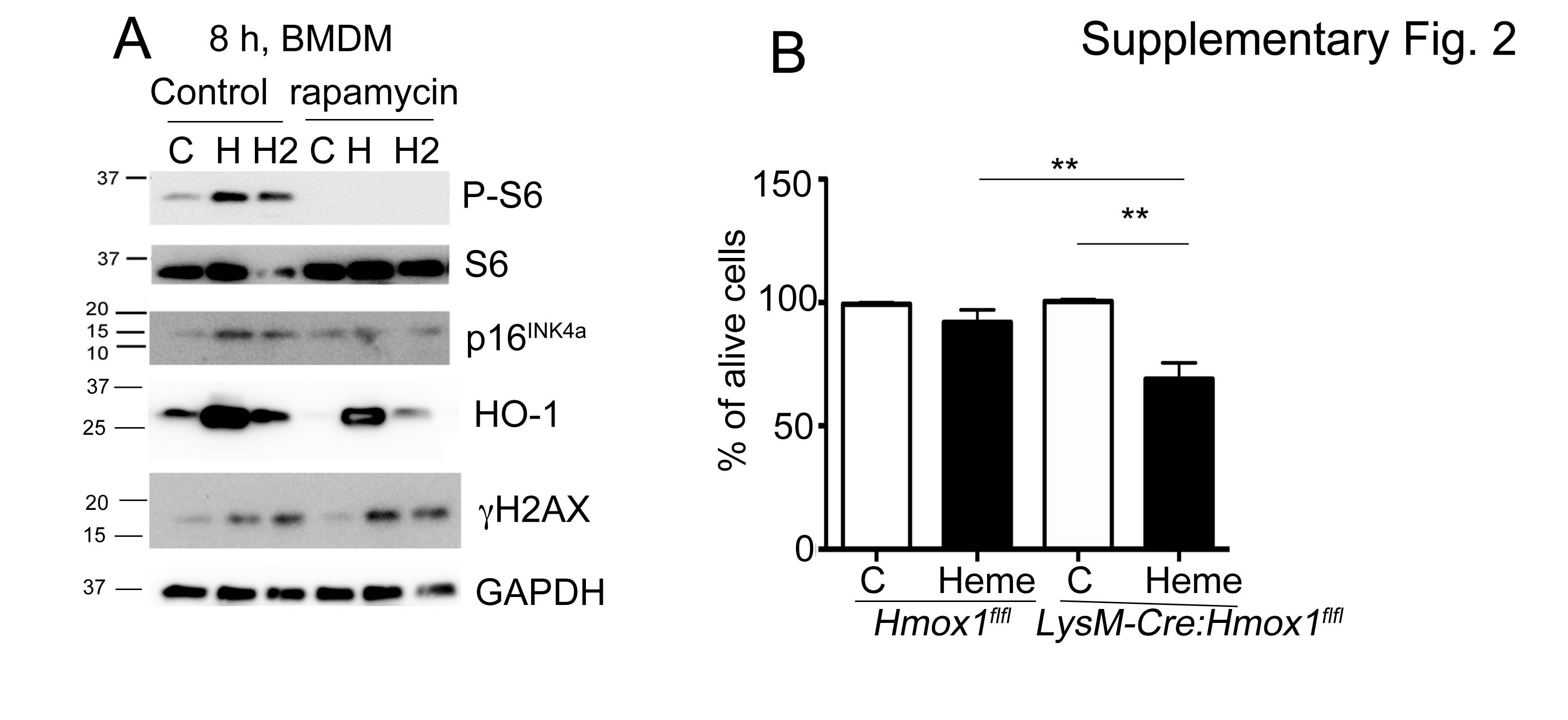

Supplement: Supplementary file 2 — Supplementary Figure 2 [file 41419_2019_1342_MOESM2_ESM.jpg]
