# Supplementary material for: Heme detoxification by heme oxygenase-1 reinstates proliferative and immune balances upon genotoxic tissue injury
Source: Cell Death Dis. 2019 Jan 25;10(2):72. doi: 10.1038/s41419-019-1342-6 (PMC6347604; doi:10.1038/s41419-019-1342-6)
Supplement: Supplementary file 3 — Supplemental figure legends [file 41419_2019_1342_MOESM3_ESM.docx]

**Supplementary Figure legends:**

**Supplementary Figure 1. Role of HO-1 in controlling expression of genes encoded in INK4 locus. A-B.** Real time PCR with primers against p16^INK4a^, p27 or p21 was performed on the cDNA of fibroblasts isolated from *Hmox1*^+/+^, *Hmox1*^+/-^ and *Hmox1^-/-^* animals. n=3 replicates. *p<0.05. **C**. Real time PCR with primers against p16^INK4a^ was performed on the cDNA of fibroblasts transfected with scramble siRNA or siRNA against p16^INK4a^. Cells were transfected with siRNA for 48h and then seeded for BrdU proliferation assay.

**Supplementary Figure 2. Role of heme in controlling cell cycle and DNA damage. A.** BMDM were treated with rapamycin prior addition of heme (H) or H_2_O_2_ (H2). Western blot was performed in the lysates of these cells and probed with antibodies against P-S6, S6, p16^INK4a^, HO-1 and H2AXγ. GAPDH was used as a loading control. These data are representative of n=3 independent experiments. **B**. BMDM isolated from *Hmox1^flfl^* and *LysM-Cre:Hmox1^fl/fl^* mice were treated with heme or left untreated (C) for 24h. Survival of cells was measured by crystal violet. % of survival is shown. **p<0.01.
